# The Level of Adherence to the ESPEN Guidelines for Energy and Protein Intake Prospectively Influences Weight Loss and Nutritional Status in Patients with Cancer

**DOI:** 10.3390/nu15194232

**Published:** 2023-09-30

**Authors:** Michail Kipouros, Konstantina Vamvakari, Ioanna Panagiota Kalafati, Iliana Evangelou, Arezina N. Kasti, Rena I. Kosti, Odysseas Androutsos

**Affiliations:** 1 Laboratory of Clinical Nutrition and Dietetics, Department of Nutrition-Dietetics, School of Physical Education, Sport Science and Dietetics, University of Thessaly, 42132 Trikala, Greece; mihalis.kip@gmail.com (M.K.); kvamvakari@yahoo.com (K.V.); ikalafati@uth.gr (I.P.K.); iliana.evgl@gmail.com (I.E.); rkroukou@gmail.com (R.I.K.); 2 Department of Nutrition & Dietetics, School of Health Science & Education, Harokopio University, 17676 Kallithea, Greece; 3 Department of Nutrition & Dietetics, Attikon University General Hospital, 12462 Athens, Greece; kastiare@yahoo.gr

**Keywords:** prospective study, nutritional status, cancer, ESPEN recommendations, PG-SGA

## Abstract

Nutrition therapy aims to prevent weight loss and its health consequences in patients with cancer. The aim of this study was to assess Greek patients’ adherence to the ESPEN guidelines for oncology patients and its prospective effect on their body weight (BW) and nutritional status. In total, 152 patients with cancer were recruited from the Attikon University Hospital, Greece, and provided data in 2019 (baseline) and 2020 (follow-up) (drop-out rate = 28.3%). Nutritional status was assessed with the PG-SGA questionnaire. Patients were categorized based on whether they adhered at least to the minimum ESPEN-recommended intakes of energy (≥25 kcal/kg/day) or protein (≥1.0 g/kg/day) or not. On average, patients did not adhere to ESPEN guidelines for energy and protein intake. Most patients meeting the minimum recommendations had an improvement of their nutritional status at follow-up and increased their BW compared to those not meeting them. All patients with head, neck, and spinal cancer who met the minimum recommendations for energy intake improved their nutritional status at follow-up. This study showed that consuming at least the minimum amounts of protein and energy recommended by ESPEN may prevent from weight loss and improve nutritional status; however, the exact amounts need to be personalized.

## 1. Introduction

The occurrence of tumor diseases is the result of the interaction of genetic and environmental factors (including dietary factors). The prevalence of cancer survivors is increasing due to innovations in cancer screening (early detection) and treatment [1]. Evidence from experimental and observational studies suggests that factors such as diet, physical activity, and obesity may influence recurrence risk and overall survival after cancer diagnosis [2]. In Western countries, the 5-year survival rate for all cancers is approximately 68% [1,3] and the number of cancer survivors in the USA is projected to increase to 26.1 million by 2040 [1].

The nutritional status of patients with cancer can vary due to a variety of modifiable and non-modifiable factors. Malnutrition is a common feature in patients with cancer, which can adversely affect clinical outcomes and lead to prolonged hospital stays [4,5]. The most common causes are increased energy and protein requirements due to the catabolic and physiological effects of cancer cachexia, inadequate food intake, and reduced physical activity [5]. Nutritional problems in patients should be considered on a continuum, ideally from the first signs and symptoms (i.e., degree of depletion of energy stores and body protein) through to pre-cachexia, cachexia, and refractory cachexia. While patients with refractory cachexia are less likely to react to nutritional therapy, the pre-cachexia and cachexia stages afford distinct and obvious windows of opportunity for nutritional intervention, with significant influence on clinical outcomes such as mortality [6]. However, it is well known that the effectiveness and impact of any nutritional intervention depends on the timing of support, with early intervention achieving the best results [7]. Nutrition therapy aims to prevent weight loss and to avoid or lessen the effects of weight loss in patients with cancer. According to a recent literature review, the dose of amino acids capable of supporting a positive protein balance in cancer patients might be close to 2 g/kg/day [8]. For clinicians, such as doctors, dietitians, and nurses, the European Society for Parenteral and Enteral Nutrition (ESPEN) and the American Society for Parenteral and Enteral Nutrition (ASPEN) have established evidence-based guidelines for nutrition management and specifically for weight loss prevention in cancer patients. ESPEN and ASPEN advise ambulant cancer patients to consume, respectively, 1 g/kg/day of protein (if possible, up to 1.5 g/kg/day) and 25 to 30 kcal/kg/day of energy [9].

Given the above and as a consequence, adherence to ESPEN recommendations for energy and protein intake by patients with cancer was hypothesized to have a positive impact on nutritional status. The aim of the present study was to investigate for the first time the adherence of Greek patients with cancer to the ESPEN recommendations for energy and protein intake and its association with changes in body weight and nutritional status.

## 2. Materials and Methods

### 2.1. Participants and Experimental Design

This prospective study was conducted at the Attikon University Hospital, Greece. Data were collected at two time periods with a 6-month gap, i.e., April–October 2019 (baseline) and February–March 2020 (follow-up). In total, 152 patients with cancer were recruited [10]. The following inclusion criteria were applied: patients were hospitalized or followed cancer treatment at the Attikon University Hospital, Greece; all types and stages of cancer were included. Exclusion criteria were as follows: patients’ age < 18 years; bedridden patients in advanced stage of the disease who were unable to carry out the measurements; patients facing difficulties in communication. All patients provided written informed consent prior to their enrollment in the study. The study was approved by the Bioethics Committee of the hospital (E.B.D. 315). All comparisons were made between the 109 patients who provided complete data at both time points (drop out rate = 28.3%).

### 2.2. Sociodemographic and Clinical Factors

All surveys were filled out through patient interviews conducted by well-trained research assistants. The sociodemographic questionnaire was used to collect data on a variety of factors, including age, gender, nationality, degree of education, marital status, residence, and occupation. Additionally, it covered the health habits of the patients, such as smoking, drinking, sleep (both quantity and quality), sedentary behavior (time spent on screen activities), and physical activity. Medical records were consulted for clinical characteristics, such as cancer type, cancer stage, type of treatment, comorbidities, and medications or dietary supplements. Breast, lung, gastrointestinal (GI) tract and colorectal, head, neck, and spinal, and other types of cancer (i.e., prostate, endometrium, ovaries, liposarcoma, lymphoma, thyroid gland, and gastrointestinal tract) were the categories into which cancers were divided.

### 2.3. Anthropometric Measurements

Using an SECA 220 scale, the body weight (BW) was measured to the nearest 100 g, the majority of the time in the morning; patients were dressed in light clothing and without shoes. To the nearest 0.1 cm, height was measured without shoes using a stadiometer.

### 2.4. Assessment of Nutritional Status

In oncology and other chronic catabolic illnesses, the Patient-Generated Subjective Global evaluation (PG-SGA) is the premier interdisciplinary patient nutritional evaluation validated tool. A variety of variables were evaluated, including the patient’s history of weight loss, the existence of symptoms, their nutritional intake, if they needed tube feeding or other forms of supplementation, the assessment of additional metabolic needs, and their physical condition. Patients were divided into three categories using the PG-SGA: category A (“good nutritional status”), category B (“moderately malnourished”), and category C (“severely malnourished”) (Jager-Wittenaar et al., 2017 [11]. The PG-SGA items’ internal consistency was satisfactory (Cronbach’s alpha = 0.722) [12]. Individuals who worsened their nutritional status and changed from category A to B or C during the 6 months were defined as “deterioration”, those who remained in the same category were defined as “no change”, while those who had a better nutritional status at follow-up and changed category (i.e., from B to A) were categorized as “improvement”. Finally, weight change was calculated as the fraction of initial weight minus final weight to initial weight and multiplied by 100.

### 2.5. Dietary Intake Analysis

Dietary intake was assessed through 24 h recalls, in which volunteers were asked to detail all foods and drinks consumed in the previous 24 h period. The reference period was the time from the moment of awakening to the next awakening. The 24 h recall was chosen over other methods (e.g., semi-quantitative food frequency questionnaire, 3-day dietary intake diary) as it requires limited time for its completion, does not require specialist knowledge of nutrition on the part of the volunteer, limits the workload of the volunteers (compared to the completion of 3-day diaries), and allows the recording of the simultaneous consumption of food and drink combinations and the context of the dietary recruitment (place, time, time, co-actors, and parallel activities) [13,14].

For each volunteer in the study, one recall was performed at two time periods. Furthermore, the recalls were carried out after participants’ consent but unannounced and thus did not allow volunteers to modify their dietary intake pending the dietary assessment [13]. The recalls were conducted by dietitians, who were trained well in this method. The same dietician made all the recalls for each volunteer. In addition, the research assistants were not aware of the weight status of the volunteers to ensure impartiality in data recording and analysis [14].

Recording was performed using the multiple-pass 24 h recall [14]. According to the above method, the time, type, and quantity of food and/or drink consumed was recorded on the first pass. On the second pass, the recaller would repeat the above, and ask the volunteer for additional information about the place of food intake, parallel activities, and possible accomplices. On the third pass, the research assistant would repeat the information collected and ask for clarification if needed. Common measures such as cups, soup spoons, grams, or the palm of the hand were used to facilitate recording the amount consumed.

After recording their dietary intake, the volunteers were asked to report the total amount of water they consumed during the reference period, whether they were taking a dietary supplement, and finally whether they were currently following a calorie-deficient diet. The last question was added to distinguish whether the volunteers had voluntarily reduced their dietary intake.

The 24 h recall data were analyzed for energy and macronutrients using the Soma Nutri Version 5.3.0 nutritional software (Soma Nutri; Serinth Technologies, Athens, Greece).

### 2.6. European Society for Parenteral and Enteral Nutrition Recommendations

The ESPEN recommends consuming 25–30 kcal/kg BW per day for calorie intake and 1.0–1.5 g/kg BW per day for protein [9]. To determine if reaching the minimum recommended intake amounts of macronutrients could affect weight loss, the minimum ESPEN guidelines for energy (≥25 kcal BW/kg/day) and protein (≥1.0 g/kg BW/day) were utilized as reference values for this study. Patients were divided into two groups: patients meeting and not meeting the ESPEN-recommended intakes for energy and protein at baseline and follow-up.

### 2.7. Statistical Analysis

The normality of the distribution of the variables investigated was evaluated by using the Kolmogorov–Smirnov test. Continuous variables are presented as median values (interquartile range (IQR)) while the categorical variables as relative frequencies (%). The non-parametric test Mann–Whitney was used to compare differences between two independent groups, while the Wilcoxon signed-rank test was performed to assess differences between the two time-points within the same sample. Chi-square tests were applied to assess independence between categorical variables. The level of statistical significance was set in all analyses at α = 0.05. All analyses were performed with the statistical package IBM SPSS Statistics, version 24 (IBM Corp., Armonk, NY, USA).

## 3. Results

The study included 109 participants (follow-up) (out of the 152 people who participated in the initial measurements). Therefore, 71.7% of the original participants agreed to participate again. Of the 28.3% of individuals who were not reassessed (drop-out rate), 17.8% could not be reached by phone, 9.8% of individuals were deceased, and 0.7% had a lack of interest. The majority of patients were women (58.7%). The median age was 60 years and most of the participants stated a higher level of education (64.1%). The most prevalent types of cancer were head, neck, and spinal (30.3%) and GI tract and colorectal cancer (24.2%), while 60.6% of them were in cancer stage IV. The most common sites of metastasis were GI tract and colorectal (46.7%), head, neck, and spinal (26.7%), lung and breast (14.1%). Almost all patients received chemotherapy (97%) (Table 1).

**Figure 1:**
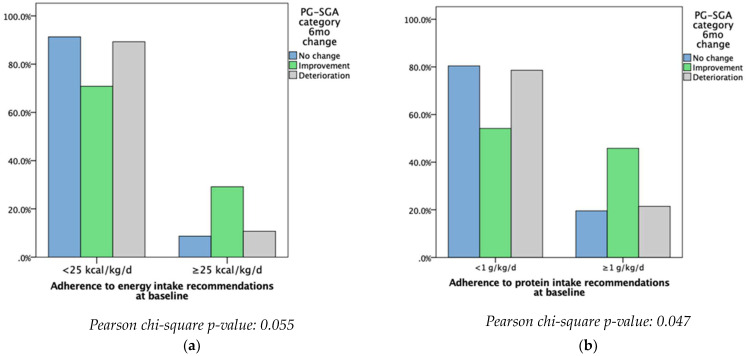
Adherence to protein and energy recommendations at baseline and change in nutritional status at follow-up. (**a**) Adherence to energy intake recommendations at baseline; (**b**) adherence to protein intake recommendations at baseline.

Patients meeting and not meeting the ESPEN recommended intakes were then compared to the PG-SGA categories. Patients who did not meet at least the minimum ESPEN energy recommendations were more likely to have deteriorated or seen no change in their nutritional status at baseline. However, most of the patients who met at least the minimum ESPEN energy recommendations had an improvement in their nutritional status (*p* = 0.055). Patients who did not meet at least the minimum ESPEN protein recommendations were more likely to see no change or a deterioration in their nutritional status, while the majority of those who met at least the minimum recommendations reported improvements in terms of nutritional status (*p* < 0.047) (Figure 1).

The effect of adherence to ESPEN guidelines on nutritional status was then assessed separately in each cancer type category. All patients with head, neck, and spinal cancer meeting at least the minimum ESPEN energy recommendations presented with an improved (according to PG-SGA) nutritional status (*p* = 0.006), while all patients who did not meet at least the minimum ESPEN energy recommendations were more likely to see no change or a deterioration of their nutritional status (*p* = 0.006). All patients with lung cancer meeting at least the minimum ESPEN protein recommendations experienced improvement or no change in their nutritional status compared to those not meeting at least the minimum recommendations, out of whom no one improved their nutritional status (*p* = 0.065). The effect of adherence to ESPEN energy recommendations was mixed among patients with GI tract and colorectal cancer, since both groups of patients experienced improvement and deterioration of their nutritional status (*p* = 0.053). No other differences were observed for the rest of the cancer types (Table 2).

**Figure 2:**
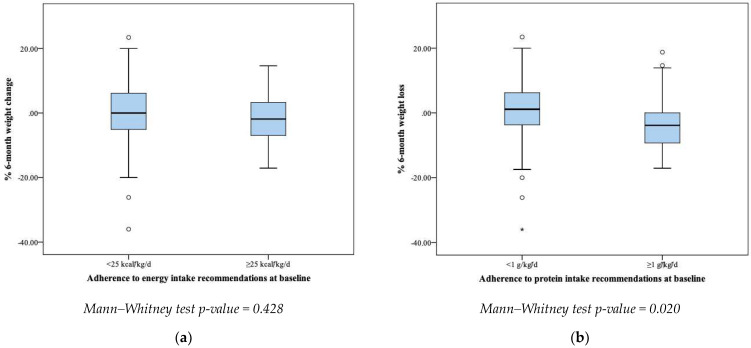
Adherence to protein and energy recommendations at baseline in correlation with weight change. (**a**) Adherence to energy intake recommendations at baseline; (**b**) adherence to protein intake recommendations at baseline.

Meeting at least the minimum ESPEN energy recommendations did not significantly affect the % 6-month weight change (*p* = 0.428). On the contrary, patients meeting at least the minimum ESPEN protein recommendations significantly increased their BW within 6 months compared to those who did not meet the recommendations (*p* = 0.020) (Figure 2).

Table 3 outlines the energy and protein intake, and BW for all patients (N = 109) at each timepoint. Both at baseline and at follow-up, this group of Greek patients did not meet at least the minimum recommendations for energy intake, reporting approximately half the calories recommended by ESPEN at both times. In terms of protein intake, it was at better levels but still did not meet the minimum recommendations. Overall, energy and protein intake were not significantly different between the two time points and BW was not significantly changed (Table 3).

Table 4 summarizes the median energy and protein intake at baseline and the percentage of weight loss at follow-up of patients meeting and not meeting at least the minimum ESPEN-recommended intakes for energy and protein at baseline. The energy and protein intake at baseline was significantly higher in those who met at least the minimum recommendations than those who did not (*p* < 0.001). The percentage of patients who lost weight at follow-up was similar between patients adherent and patients non-adherent to energy recommendations. On the other hand, a lower percentage of patients adhering to protein recommendations lost weight during this 6-month period compared to non-adherent patients (22.2% vs. 52.1%). Patients meeting at least the minimum ESPEN-recommended intakes for energy who lost weight had a lower % weight loss than those not meeting them (*p* = 0.701). Surprisingly, % weight loss was higher in patients adherent compared to patients non-adherent to protein recommendations (*p* = 0.122). However, both results were not statistically significant.

## 4. Discussion

The main findings of this study relate to the change in BW and in nutritional status according to the PG-SGA and how meeting or not meeting at least the minimum ESPEN recommendations for energy and protein intake may affect these changes. It should be noted that this is one of the few studies, at least for the Greek population, in which it even appeared that most Greek patients with cancer do not meet at least the minimum ESPEN recommendations for energy and protein intake, highlighting the need for more intense interventions by healthcare professionals. In more detail, patients who did not meet at least the minimum ESPEN energy recommendations were more likely to have deteriorated or seen no change in their nutritional status at baseline. However, most of the patients who met at least the minimum ESPEN energy recommendations had an improvement in their nutritional status from baseline to follow-up. There was a similar pattern in protein intake, as patients who did not meet at least the minimum ESPEN protein recommendations were more likely to have no change or a deterioration in their nutritional status, while the majority of those who met at least the minimum recommendations reported improvements in terms of nutritional status. Another important finding is the fact that patients meeting at least the minimum ESPEN protein recommendations significantly increased their BW within 6 months compared to those who did not. Finally, all patients with head, neck, and spinal cancer meeting at least the minimum ESPEN energy recommendations presented with an improved nutritional status, while all patients who did not meet at least the minimum ESPEN energy recommendations were more likely to have no change or a deterioration in their nutritional status.

Regarding the anthropometric measurements, in the present study the mean value of the participants’ BW was 72.8 kg, i.e., 1.8 kg higher than the initial measurements (71.0 kg in 2019). This is mainly attributed to the fact that about half of the subjects had completed their part of the treatments and were in recovery, whereas at the initial measurements all patients were following at least one treatment regimen. The average BMI (25.6 kg/m^2^) falls within the lower limits of overweight according to the World Health Organization. In most prospective studies, such as Dotan and colleagues (mean BMI: 26.0 kg/m^2^) and Westby and colleagues (mean BMI: 28.3 kg/m^2^), the BMI of patients with cancer was also reported to be in the lower limits of excess weight, and to extend to the limits of normal weight in fewer studies, such as Barthelemy and colleagues (mean BMI: 23.7 kg/m^2^) [15,16,17]. However, it should be emphasized that BMI focuses on identifying excess weight (not enough information on body composition), so it may coexist with malnutrition/cachexia. After all, while it is often assumed that overweight or even obese cancer patients are eating normally, in reality there may have severe muscle wasting [18]. Finally, recent studies on cancer patients have shown an inverse relationship between BMI and mortality, called the “obesity paradox”, which suggests that overweight/obese oncology patients have better prospects of survival [19,20].

PG-SGA was used to further assess the nutritional status of the patients. Between the two time periods, there was a decrease in the proportion of patients with malnutrition. In 2019, 13.8% (15/109) of patients were considered moderately malnourished and 26.6% (29/109) severely malnourished. These percentages were found to change within the next 6 months of the study, as only 8.3% (9/109) and 17.4% (19/109) of patients were moderately and severely malnourished, respectively. These results were expected considering that the initial measurements were taken exclusively from hospitalized patients or from patients who attended the hospital at regular intervals due to their treatments (mainly chemotherapy), while in the later measurements a significant percentage of these patients had completed their treatment and were recovering. Through chemotherapy (a treatment that almost all patients have had) a full recovery can be achieved, even if the cancer has spread. Therefore, patients who were in the recovery phase were likely to have taken actions to improve their nutritional status. This is also confirmed by information collected on the nutritional status of patients through 24 h recalls. In line with the above, Marshall and colleagues’ research showed that there was an equal decrease in the prevalence of malnutrition between two points in time, with 31% of malnourished people in 2012 reaching 26% in 2014 [21]. However, in another prospective study, the proportion of malnourished patients appeared to increase, with 66.7% of patients found to be malnourished reaching 87.7% within two years [22]. This discrepancy can be explained by differences in patients, including different types of cancer, cancer stages, and differences in treatment and methods of assessing malnutrition. The PG-SGA, finally, showed that 73 patients out of a total of 109 had a score greater than or equal to 9 and required immediate nutritional support with a high risk of death, while the following year only 44 patients were in the same position. The prospective study by Dotan and colleagues confirms these results [17]. Furthermore, according to the categories created for the purposes of this study (deterioration, no change, and improvement), it appeared that patients who did not adhere at least to the minimum ESPEN recommendations for energy and protein either deteriorated or stagnated in their nutritional status. On the other hand, the majority of those who followed at least the minimum recommendations experienced an improvement in their nutritional status. Non-compliance with at least the minimum ESPEN recommendations also led to poor nutritional adequacy, as has been observed in similar studies [23,24].

All patients with head, neck, and spinal cancer meeting at least the minimum ESPEN energy recommendations had an improvement (according to PG-SGA) in the nutritional status and, consequently, weight loss. However, studies on head and neck cancer patients revealed that even consuming the minimum suggested amounts of protein and energy did not stop individuals with head and neck cancer from losing weight and skeletal muscle [23,25]. Patients with GI tract and colorectal cancer experienced both improvements and deteriorations in their nutritional status as a result of following at least the minimum ESPEN energy recommendations. These results provide evidence that simply meeting at least the minimum recommended levels of oral intake for protein and energy does not prevent patients with any type of cancer from experiencing malnutrition and weight loss. Cancer-related malnutrition is the result of a complicated combination of negative energy and protein balance, systemic inflammation syndrome, hypoanabolism, and tumor or inflammation-derived hypercatabolism [5,26,27]. More research is needed to determine the energy and protein requirements of oncology patients, as the requirements for weight maintenance may vary depending on the level of metabolic changes in various cancer types and treatment regimens; the decisions in nutritional care process need to take into account the individual needs of each patient, thus leading to a personalized approach.

Meeting at least the minimum ESPEN energy recommendations did not significantly affect % weight change, while patients meeting at least the minimum ESPEN protein recommendations significantly increased their body weight within 6 months compared to those not meeting at least the minimum recommendations. Patients not meeting at least the minimum ESPEN energy recommendations at baseline were given nutritional counselling with or without the use of oral or artificial (enteral or parenteral) nutritional supplements, and generally received more nutritional care than those who met at least the minimum recommendations. Therefore, after 6 months no weight loss was detected due to the intensive care provided by hospital dieticians. As expected, the majority of patients not meeting at least the minimum ESPEN protein intake lost weight, which is a common observation according to other studies [23,25]. Cancer patients should be monitored frequently and by a multidisciplinary team to meet their nutritional needs, prevent weight loss and loss of muscle mass, and maintain functional capacity and/or quality of life [28,29]. Dietitians/Nutritionists are trained in the nutritional analysis of dietary intake and the design of diets, and can provide alternatives for adequate energy and, primarily, protein intake through dietary sources and supplements, while also suggesting other strategies (e.g., artificial nutrition) for adequate feeding and coverage of nutritional needs. Oral nutrition is always a priority in clinical practice since it increases patient autonomy and quality of life. Oral feeding is not always possible (for example, due to decreased digestive system functionality, limited capacity to swallow, etc.). As a result, the patient should be provided with the proper artificial nourishment to satisfy their demands. Therefore, it is recognized that multidisciplinary monitoring (both medical doctors and nurses, as well as dietitians/nutritionists) for early and frequent nutritional intervention is of great importance in oncology and a key factor for successful treatment and recovery [29].

At follow-up, the percentage of patients who lost weight was similar between patients adherent and patients non-adherent to energy recommendations. This may be due to the fact that 60.6% of the cancer patients in our sample were stage IV, so even those who met at least the minimum ESPEN energy recommendations may have been driven to weight loss due to the advanced disease stage and increased catabolism. On the other hand, being adherent to the protein intake guidelines resulted in a better BW status and lower rates of weight loss. However, the percentage of BW loss was higher in patients adherent compared to patients non-adherent to protein recommendations. This seems unexpected; however, the small sample size of the study and the fact that the majority of patients were stage IV may explain this condition. However, both aforementioned results were not statistically significant. The inflammatory processes that are enhanced during cancer development, especially in stage IV patients, have been linked to anorexia combined with reduced response to overall treatment, thus aggravating disease prognosis [30]. According to McGovern et al., another definition of cancer cachexia could be “disease-related inflammation with malnutrition” in an attempt to highlight the need for early detection and intervention in patients with cancer at risk of malnutrition [31]. Moreover, a personalization of nutritional therapy to the needs of advanced-stage cancer patients might be necessary to improve their nutritional status.

In the clinical setting, there are various barriers prohibiting patients from consuming high amounts of protein and energy during or after a hospital stay. The present study is one of the few in this field that have been conducted in real-life conditions. The dietitians who collected the primary study data reported many barriers to implementing dietary advice in patients with cancer. Dietitian-reported non-symptom-related barriers such as poor motivation, conflicting advice, food preference restrictions, and communication difficulties were very common among the patients. Symptom-related barriers were mainly side effects from chemotherapy/radiotherapy such as lower gastrointestinal symptoms, swallowing difficulties, fatigue, anorexia, nausea and vomiting, taste changes and dry mouth, pain, anxiety, and depression. In fact, all of the above are confirmed by a recent study that described these barriers [32]. A multidisciplinary team is required, and involvement from other practitioners, such as specialists in symptom control, psychosocial therapy, and social work, may each play a role in resolving the numerous barriers to nutritional intervention identified.

Strengths of this study include the prospective nature of the design. In addition, there are very limited studies that have investigated the adherence to the ESPEN protein and energy recommendations in patients with cancer. Still, the findings of this study should be interpreted under the light of its limitations. The study sample was relatively small and obtained from a single hospital in Athens, so the results may not be applicable to the entire Greek population of cancer patients. Finally, regarding the 24 h recalls, one recall was collected from each patient which may limit the representativeness of patients’ energy and protein intake. However, all recalls (baseline/follow-up) were from weekdays so some comparison could be made and according to a recent study the 1-day method is a viable method for determining average intakes of frequently ingested dietary components [33].

## 5. Conclusions

Maintaining a healthy nutritional status in patients with cancer is still a major therapeutic problem that is linked to clinical outcomes. The findings of the present study showed that, on average, Greek patients with cancer do not meet the minimum recommended energy and protein intakes by ESPEN. Consuming at least the minimum amounts may be beneficial for patients with cancer to stop losing weight, especially for certain cancer types. Adequate energy and protein intake is an important part of the nutritional care process in patients with cancer. The exact quantity of energy and protein needed for the prevention of weight loss and deterioration of nutritional status may vary among patients, and therefore a personalized approach (e.g., based on cancer stage) and an early detection and intervention should be applied to meet each patient’s individual needs.

## Figures and Tables

**Table 1:** Characteristics of study participants (N = 109).

Followed-up (%)	71.7
Non-compliance rate (%)	
- Deceased	9.8
- Missed/No interest	18.5
Sex, men (%)	41.3
Age (years)	60.1 (12.21)
Education level	
- Primary (%)	22.3
- Secondary (%)	13.6
- Higher (%)	64.1
Marital status	
- Married/with partner (%)	68.8
Cancer stage	
- I (%)	9.1
- II (%)	17.2
- III (%)	13.1
- IV (%)	60.6
Cancer types	
- Head, neck, and spinal (%)	30.3
- GI tract and colorectal (%)	24.2
- Lung (%)	14.1
- Breast (%)	14.1
- Other types * (%)	17.2
Migration	
- Head, neck, and spinal (%)	26.7
- GI tract and colorectal (%)	46.7
- Lung (%)	20.0
- Breast (%)	0.0
- Other types * (%)	6.7
Types of treatment	
- Chemotherapy (%)	97.0
- Radiotherapy (%)	58.4
- Surgery (%)	57.4
- Immunotherapy/Biological therapy (%)	42.6
- Hormonotherapy (%)	9.9
- Transplantation (%)	0.0

* Other types: prostate, endometrium, ovaries, liposarcoma, lymphoma, thyroid gland, and gastrointestinal tract.

**Table 2:** Adherence to ESPEN guidelines at baseline and change in nutritional status per cancer type category.

(%)	Patients Meeting at Least the Minimum ESPEN Energy Recommendations (≥25 kcal/kg/Day) (N = 15)	Patients Not Meeting at Least the Minimum ESPEN Energy Recommendations (<25 kcal/kg/Day) (N = 85)	p	Patients Meeting at Least the Minimum ESPEN Protein Recommendations (≥1 g/kg/Day) (N = 27)	Patients Not Meeting at Least the Minimum ESPEN Protein Recommendations (<1 g/kg/Day) (N = 73)	p
Head, neck, and spinal						
No change	34.5	0	0.006	31	3.6	0.097
Improvement	10.4	6.9		6.9	10.3	
Deterioration	48.2	0		37.9	10.3	
GI tract and colorectal cancer						
No change	63.6	0	0.053	59.1	4.5	0.180
Improvement	22.6	4.6		18.3	9.1	
Deterioration	4.6	4.6		4.5	4.5	
Lung						
No change	38.5	23.1	0.928	23.1	38.5	0.065
Improvement	7.7	7.7		0	15.3	
Deterioration	15.3	7.7		23.1	0	
Breast						
No change	33.3	0	0.466	25	8.3	0.539
Improvement	25	0		8.3	16.7	
Deterioration	33.3	8.4		25	16.7	
Other						
No change	38.5	7.6	0.263	38.4	7.7	0.658
Improvement	15.4	15.4		23.1	7.7	
Deterioration	23.1	0		23.1	0	

Values presented as %. *p*: Pearson chi-square *p*-value.

**Table 3:** Energy and protein intakes and BW in patients with cancer (N = 109) measured at baseline and follow-up.

	Baseline	Follow-up	*p*
Energy, kcal/kg/day	15.26 (10.57)	15.73 (7.09)	0.978
Protein, g/kg/day	0.71 (0.58)	0.69 (0.43)	0.213
BW, kg	70.50 (22)	73 (22)	0.837

Values presented as median (Interquartile Range (IQR)). *p*: Wilcoxon *p*-value.

**Table 4:** Weight loss, and energy and protein intakes of patients meeting and not meeting at least the minimum ESPEN recommendations.

	Patients Meeting at Least the Minimum ESPEN Energy Recommendations (≥25 kcal/kg/day) (N = 15)	Patients Not Meeting at Least the Minimum ESPEN Energy Recommendations (<25 kcal/kg/day) (N = 85)	p
Age (years)	59.00 (20)	61.50 (14)	0.244
Sex, men (%)	40.0	43.5	0.799
Energy intake at baseline, kcal/kg/day	28.82 (11.99)	13.89 (8.62)	2.8 × 10^7^
Patients losing weight at this energy intake	6	38	
% 6-month weight loss of these patients	3.97 (11.14)	6.42 (7.09)	0.701
	**Patients Meeting at Least the Minimum ESPEN Protein Recommendations (≥1 g/kg/day) (N = 27)**	**Patients Not Meeting at Least the Minimum ESPEN Protein Recommendations (<1 g/kg/day) (N = 73)**	**p**
Age (years)	59.00 (17)	62 (14)	0.239
Sex, men (%)	48.1	41.1	0.650
Protein intake at baseline, g/kg/day	1.06 (0.39)	0.55 (0.41)	1.9 × 10^14^
Patients losing weight at this protein intake	6	38	
% 6-month weight loss of these patients	12.1 (11.37)	6.20 (6.59)	0.122

Mann–Whitney test and chi-square test *p*-values are presented for continuous and categorical variables, respectively.

## Data Availability

The data presented in this study are available on request from the corresponding author. The data are not publicly available due to data protection issues.
